# Human brain pericytes protect the blood–brain barrier from triple‐negative breast cancer cells while promoting tumor aggressiveness

**DOI:** 10.1002/ccs3.70070

**Published:** 2026-05-03

**Authors:** Eloise Happernegg, Marine Carroué, Julien Cicero, Lucie Dehouck, Fumitaka Shimizu, Takashi Kanda, Chann Lagadec, Fabien Gosselet, Xuefen Le Bourhis, Maxime Culot, Robert‐Alain Toillon, Caroline Mysiorek

**Affiliations:** ^1^ University of Lille CNRS, Inserm, CHU Lille, Institut de Recherche Contre Le Cancer de Lille, UMR9020 ‐ UMR‐S 1277 ‐ Canther Cancer Heterogeneity, Plasticity and Resistance to Therapies Lille France; ^2^ University Artois UR 2465, Laboratoire de la Barrière Hémato‐Encéphalique (LBHE) Lens France; ^3^ Department of Neurology and Clinical Neuroscience Graduate School of Medicine Yamaguchi University Ube Japan; ^4^ Department of Neurology Neuromuscular Center Yoshimizu Hospital Shimonoseki Japan; ^5^ GdR2082 APPICOM‐ « Approche Intégrative Pour Une Compréhension Multi‐Échelles de La Fonction Des Protéines Membranaires » Paris France

**Keywords:** blood–brain barrier, brain metastasis, breast cancer, microenvironment, pericytes

## Abstract

The majority of breast cancer‐related deaths result from metastatic progression, especially brain metastasis, associated with the poorest prognosis. Patients with triple‐negative breast cancer (TNBC) are particularly prone to developing brain metastases. In this context, the blood–brain barrier (BBB) plays a pivotal role; however, the contribution of brain pericytes in mediating interactions between TNBC cells and the brain endothelial cells (ECs) remains unclear. To investigate the role of pericytes in modulating these interactions, we used a syngeneic human in vitro BBB model, composed of human brain‐like endothelial cells (hBLECs) co‐cultured with human brain pericytes (hBPs). Our findings demonstrate that hBPs secretions significantly reduce TNBC cell adhesion to hBLECs and protect the endothelium from TNBC cell‐induced damage, both under normoxic and hypoxic conditions. Interestingly, we found that hBPs promote TNBC cell migration, invasion, and increase clonogenicity. Together, these findings uncover a dual role of brain pericytes in TNBC brain metastasis: they protect ECs and maintain BBB integrity against TNBC cells while simultaneously promoting tumor aggressiveness. Understanding this balance between protective and pro‐metastatic functions may provide insights for developing novel therapeutic approaches to strengthen BBB defenses and limit brain colonization in TNBC patients.

## Graphical abstract

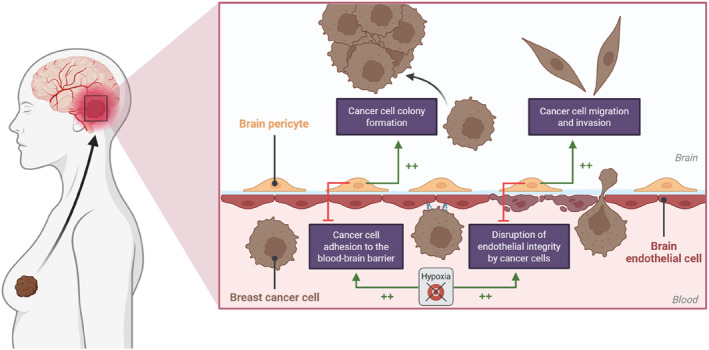

## 1 INTRODUCTION

With over two million new cases and 666,000 deaths each year, breast cancer remains the leading cause of cancer‐related mortality among women worldwide,^1^ mainly because of metastatic progression. Among the most common metastatic sites (lung, bones, liver, and brain), brain metastases occur in approximately 25% of patients with metastatic disease.^2^ Neurological complications associated with brain metastases, such as motor dysfunction, memory loss, and sensory impairments, severely impact the quality of life of patients and further worsen prognosis.^3^ A higher incidence of brain metastases is observed in certain breast cancer subtypes, including triple‐negative (32%) and HER2 (human epidermal growth factor receptor 2)‐positive tumors (31%).^4^ Defined by the absence of estrogen and progesterone receptor expression and the absence of HER2 overexpression, triple‐negative breast cancer (TNBC) has the poorest prognosis, with a median survival of less than 6 months^5^ after brain metastasis diagnosis.

The “seed and soil” theory proposed by Stephen Paget,^6^ postulates that metastatic colonization depends on the compatibility between disseminated cancer cells (“seed”) and a receptive microenvironment (”soil”). Such interactions are mediated through direct cell–cell contacts and the secretion of signaling molecules, including cytokines, growth factors, and proteases. In brain metastasis, a pivotal step is the interaction between circulating tumor cells and the blood–brain barrier (BBB), which maintains central nervous system (CNS) homeostasis.^7^ Located at brain capillaries, the BBB is a specialized structure in which endothelial cells (ECs) are in close contact with pericytes and are surrounded by astrocytic end‐feet, forming a tightly regulated barrier that restricts the passage of most cells and molecules into the brain parenchyma.^8^ This restriction is enforced by low transcytosis and by the high expression of tight junction proteins. In addition, active efflux pumps and detoxifying enzymes not only restrict the entry of potentially toxic compounds but also limit the penetration of many therapeutic agents. Nevertheless, a small fraction of cancer cells can cross the BBB and survive in the brain parenchyma, where they become protected from therapies, contributing to treatment failure. Moreover, certain conditions promote brain metastasis development, including the occlusion of brain capillaries by cancer cells and their rapid growth, which can generate hypoxic regions within the vasculature.^9^, ^10^ Although pericytes are known to be essential for BBB development and maintenance,^11^, ^12^ their contribution to TNBC brain metastasis remains poorly understood. Given their strategic location at the interface between ECs and brain parenchyma, investigating the role of pericyte in brain metastasis appears relevant, as they are already implicated in multiple CNS pathologies, including Parkinson's disease,^13^ Alzheimer's disease,^14^ ischemic stroke,^15^ or even glioblastoma.^16^

In this study, using a human in vitro BBB model consisting of human brain‐like endothelial cells (hBLECs) co‐cultured with human brain pericytes (hBPs), we demonstrate that hBPs reduce TNBC cell adhesion to hBLECs and play a crucial role in preserving the endothelial integrity by protecting hBLECs from TNBC cell‐induced damage. Furthermore, exposure to hypoxic conditions enhances TNBC cell adhesion to hBLECs; however, this effect is limited by factors secreted by hBPs. Under hypoxia, cancer‐induced disruption of endothelial integrity is effectively prevented in the presence of hBPs. In addition, we provide evidence that hBPs increase clonogenicity, migration, and invasion of TNBC cells. Taken together, these findings highlight the dual and critical role of brain pericytes in the development of TNBC brain metastases.

## 2 MATERIALS AND METHODS

### 2.1 Cell culture

All cells were cultured in humidified incubators, maintaining a controlled atmosphere of 5% CO_2_ at 37°C. All cells were routinely tested for *mycoplasma* contamination (MycoAlert™, Lonza) and confirmed negative prior to use in experiments, and short tandem repeat analysis confirmed the absence of cross‐contamination. Cell lines used were not reported as misidentified or contaminated in public databases.

#### 2.1.1 Culture of cancer cells

The human TNBC cell line MDA‐MB‐231 (female) was obtained from the American type culture collection (ATCC, HTB‐26, RRID:CVCL_0062, obtained in 2023). TNBC cells were cultured in minimum essential medium (MEM; Gibco) supplemented with 10% (v/v) heat‐inactivated Fetal Bovine Serum (FBS), 1% (v/v) non‐essential amino acids (Gibco), and 1% (v/v) penicillin‐streptomycin (Gibco).

#### 2.1.2 Human in vitro BBB model

Human CD34^+^ hematopoietic stem cells (male) were isolated from umbilical cord blood and differentiated into ECs, following the protocol described by Pedroso et al.^17^ Written informed consent was obtained from the family of the participant before the collection of CD34^+^ hematopoietic stem cells, and the protocol was approved by the French Ministry of Higher Education and Research (CODECOH, DC2011–1321). Differentiated ECs were cultured in 100‐mm Petri dishes (Corning) coated with gelatin (Sigma‐Aldrich), in EC growth medium MV 2 (ECGMV2 + Supplement mix C‐39226, PromoCell) and gentamicin (50 μg/ml; Biowest).

hBPs^18^ (female) were provided by Dr. Fumitaka Shimizu and Pr. Takashi Kanda from the Department of Neurology and Clinical Neuroscience, Graduate School of Medicine, Yamaguchi University, Ube, Japan. hBPs were cultured in 100‐mm Petri dishes (Corning) coated with collagen I (100 μg/ml, Corning), in Dulbecco's modified eagle medium (DMEM; Life Technologies) containing 4.5 g/L D‐glucose and supplemented with 10% (v/v) heat‐inactivated FBS (Sigma‐Aldrich), 1% (v/v) penicillin‐streptomycin (Gibco), and L‐glutamine (2 mM, Merck).

ECs were detached using trypsin‐EDTA solution (1X; Biowest) and seeded at a density of 70,000 cells/cm^2^ onto 0.4‐μm pore‐size filter inserts (Corning) coated with growth factor‐reduced Matrigel^®^ (Corning). In parallel, hBPs were detached using trypsin‐EDTA (1X; Biowest) and seeded at a density of 13,200 cells/cm^2^ into collagen I‐coated 12‐well plates (100 μg/ml; Corning). Filter inserts containing ECs were placed above the hBPs culture and co‐cultured for 5 days (ECGMV2, PromoCell). After this co‐culture period, ECs, now referred to as hBLECs,^19^ have acquired major BBB phenotypic and functional properties, rendering the model ready for subsequent experiments.

#### 2.1.3 Culture of human astrocytes

Human astrocytes from brain cortex (Cat. #1800, ScienCell, obtained in 2019, gender information is not provided by the vendor) were cultured in astrocyte medium (ScienCell) supplemented with 2% (v/v) heat‐inactivated FBS (Sigma‐Aldrich), 1% (v/v) astrocyte growth supplement (ScienCell), and 1% (v/v) penicillin–streptomycin (Gibco) in poly‐L‐lysine (PLL, 2 μg/cm^2^, ScienCell)‐coated T75 flasks (Sarstedt).

### 2.2 Hypoxic incubation

After 5 days of ECs co‐culture with hBPs under normoxic conditions (20% O_2_, 5% CO_2_) to induce BBB properties, hBLECs and hBPs co‐culture were transferred to a hypoxia chamber (Whitley H35 Hypoxystation) maintained at 37°C, 1% O_2_, 5% CO_2_, and 94% N_2_.

In parallel, experiments were performed under normoxic conditions and were used as controls.

### 2.3 Cancer cell adhesion assay

According to the protocol previously published,^20^, ^21^ cancer cells were first labeled with CellTracker^TM^ (2.5 μg/ml; Invitrogen). Following labeling, cells were rinsed with phosphate‐buffered saline (PBS; NaCl 8 g/L, KCl 0.2 g/L, KH_2_PO_4_ 0.2 g/L, NaHPO_4_ 2.86 g/L, pH 7.4), and subsequently incubated with EDTA solution (5 mM, 10 min, 37°C). Before TNBC cells seeding, hBLECs‐containing inserts were transferred to wells without hBPs or maintained in co‐culture with hBPs to evaluate the influence of hBPs secretions. To evaluate the effect of astrocyte secretions, prior to TNBC cells seeding, inserts with hBLECs were transferred into 12‐well plates (Corning) containing human astrocytes, which had been seeded three days earlier at a density of 300,000 cells per well (coated with PLL, 2 μg/cm^2^). All BBB adhesion experiments with the different cell types, as well as the generation of the associated conditioned media, were performed in ECGMV2 with supplements, or without supplements (basal ECGMV2) depending on the experimental conditions. TNBC cells were then detached mechanically, resuspended in MEM with 1% (v/v) FBS, seeded into the luminal compartment (40,000 cells per insert), and incubated for 3 h. The inserts were rinsed twice with MEM to remove non‐adherent cells, then fixed with 4% (w/v) paraformaldehyde (PFA, 10 min), and nuclei were counterstained with Hoechst 33258 (1 μM, 10 min). The inserts were carefully cut using a scalpel and mounted between a microscope slide and coverslip using Glycergel mounting medium (Dako). The number of TNBC cells adherent to hBLECs was manually quantified (100 fields per filter) using a fluorescence microscope (Plan Fluor 20x/0.45 air objective, Eclipse Ti‐U, Nikon) with the Nikon software (NIS element AS 4.60).

### 2.4 Permeability assay

The integrity of hBLECs was assessed by quantifying the diffusion of Lucifer Yellow, an integrity marker that poorly crosses the BBB. The inserts (all coated with Matrigel^®^, with or without hBLECs) were transferred into wells of a 12‐well plate containing 1.5 ml of Ringer‐HEPES (RH) solution (NaCl 150 mM, KCl 5.2 mM, CaCl_2_ 2.2 mM, MgCl_2_ 0.2 mM, NaHCO_3_ 6 mM, HEPES 5 mM, glucose 2.8 mM; pH 7.4; 37°C). Lucifer Yellow (50 μM; Sigma‐Aldrich) diluted in RH solution was then added to the luminal compartment. Every 20 min, inserts were transferred into new wells containing fresh RH solution. After 1 h, the contents of the luminal compartment were collected. Fluorescence from the initial solution, luminal, and abluminal compartments was measured using a microplate fluorometer (432/538 nm, Synergy H1, BioTek Instruments). Permeability was determined as previously described^21^ and expressed in × 10^−3^ cm/min.

Endothelial permeability under hypoxic conditions was evaluated by performing permeability assays within the hypoxia chamber.

### 2.5 Immunofluorescence staining

Cells were fixed either with ice‐cold methanol/acetone (1:1, 45 s) for hBLECs staining, or with 4% (w/v) PFA (10 min) for MDA‐MB‐231 and hBPs staining. Following PFA fixation, unreacted aldehyde residues were quenched by washes with glycine (100 mM, 3 × 5 min), and cells were permeabilized with a solution of Triton‐X100 (0.3% (v/v), 2 × 5 min).

After three washes with PBS, filters were incubated with blocking buffer (normal donkey serum NDS 5% (v/v), bovine serum albumin BSA 1% (w/v), 1.5 h, room temperature (RT)). Cells were then incubated with the primary antibody against Claudin‐5 (1:100, rabbit, Invitrogen, 34–1600) diluted in the blocking buffer (16 h, 4°C). After primary antibody incubation, cells were washed with blocking buffer five times, followed by incubation with the secondary antibody (Alexa Fluor^TM^ 568 anti‐rabbit, 1:2000, Invitrogen, A11036) and Phalloidin (Alexa Fluor^TM^ 647 Phalloidin, 1/40, A22287) diluted in blocking buffer (1.5 hours, RT). After four washes with PBS, nuclei were counterstained with Hoechst 33258 (1 μM, 10 min), followed by three additional washes with PBS. Samples were mounted with fluorescence mounting medium (Dako) between a slide and coverslip and observed using a Confocal Imaging Reader (BioTek Cytation C10, Agilent). The mean fluorescence intensity of cortical F‐actin (near the cell membrane) and the elongation factor of TNBC cells, defined as the ratio of cell length to cell width, were quantified using ImageJ software.

### 2.6 Cancer cell migration and invasion

MDA‐MB‐231 cells were seeded (44,000 cells/cm^2^ for migration; 71,000 cells/cm^2^ for invasion) onto 3‐μm pore‐size insert filters (Corning), pre‐coated with Matrigel^®^ (1.27 mg/ml, Growth Factor Reduced, Corning) for invasion, in Dulbecco'’s modified eagle medium (DMEM) supplemented with 0.1% (v/v) FBS. The inserts were then transferred either into empty wells containing DMEM with 0.1% (v/v) FBS or into wells containing hBPs in DMEM with 0.1% (v/v) FBS, which had been previously co‐cultured with ECs for 6 days. After 24 h (migration) or 36 h (invasion) of incubation, the inserts were fixed with 4% (w/v) PFA for 10 min. Non‐migrated or non‐invaded cells on the upper surface of the membrane were carefully removed using a cotton swab. The nuclei of cells on the lower surface were counterstained with Hoechst 33258 (1 μM, 10 min), and the filters were mounted between a microscope slide and coverslip using Glycergel mounting medium (Dako). The number of cells on the lower surface was quantified (100 fields per filter) using a fluorescence microscope (Plan Fluor 20x/0.45 air objective, Eclipse Ti‐U, Nikon) equipped with NIS‐Elements AR software (NIS element AS 4.60).

### 2.7 Direct co‐culture between cancer cells and hBPs

hBPs were seeded at a density of 3100 cells/cm^2^ on type I collagen‐coated (100 μg/ml) compartmentalized slides (Thermo Fisher Nunc^TM^ Lab‐Tek^TM^) in DMEM supplemented with 2% (v/v) FBS. Three hours later, MDA‐MB‐231 cells, labeled with CellTracker^TM^ (5‐chloromethylfluorescein diacetate; 2.5 μg/ml; Invitrogen), were seeded at a density of 3100 cells/cm^2^ in the same medium, and co‐cultured with hBPs for 48 h.

### 2.8 Collection of hBP‐conditioned medium

After 5 days of co‐culture with ECs, hBPs were separated and cultured alone for 24 h in DMEM supplemented with 0.1% (v/v) FBS. The hBP‐conditioned medium (hBP‐CM) was then collected, centrifuged (1000 rpm, 10 min, 4°C), and stored at −80°C until use.

### 2.9 Clonogenic assay

MDA‐MB‐231 cells were seeded at a density of 250 cells/mL in 6‐well plates (Corning) using DMEM supplemented with 2% (v/v) FBS (2 ml per well). Three hours post‐seeding, the medium was replaced with one of the following: a 1:1 mixture of DMEM with 2% (v/v) FBS and DMEM with 10% (v/v) FBS; a 1:1 mixture of DMEM with 2% (v/v) FBS and DMEM with 0.1% (v/v) FBS, or a 1:1 mixture of DMEM with 2% (v/v) FBS and hBPs‐CM. The medium was refreshed 2 days later, and cells were further incubated for an additional 2 days. Subsequently, the medium was replaced every 4 days with DMEM containing 2% (v/v) FBS for all conditions. After 14 days of culture, cells were washed twice with PBS, fixed with 4% (v/v) PFA for 15 min, washed twice again with PBS, stained with 0.4% (w/v) crystal violet for 30 min, and washed twice with distilled water. Representative images per condition were acquired, and colonies containing more than 50 cells were counted.

### 2.10 Statistical analyses

Data were analyzed using GraphPad Prism software version 9.5. Results are expressed as mean ± standard deviation (SD). Statistical analyses were performed on data obtained from three independent experiments. Normality of the data distribution was assessed using the Shapiro–Wilk test, and homogeneity of variances was evaluated using Bartlett's test. For comparisons between two groups, Welch's *t*‐test was used when data were normally distributed and variances were unequal, and the Mann–Whitney test was used for non‐normally distributed data. For multiple comparison analysis when data were normally distributed, one‐way ANOVA followed by Tukey's post hoc test was used when variances were equal, and Welch's ANOVA followed by Dunnett's T3 post hoc test was used when variances were unequal. For multiple comparisons with non‐normally distributed data, analysis was performed using the Kruskal–Wallis test followed by Dunn's post hoc test.

## 3 RESULTS

### 3.1 Brain pericyte secretions reduce TNBC cell adhesion to the endothelium

**FIGURE 1:**
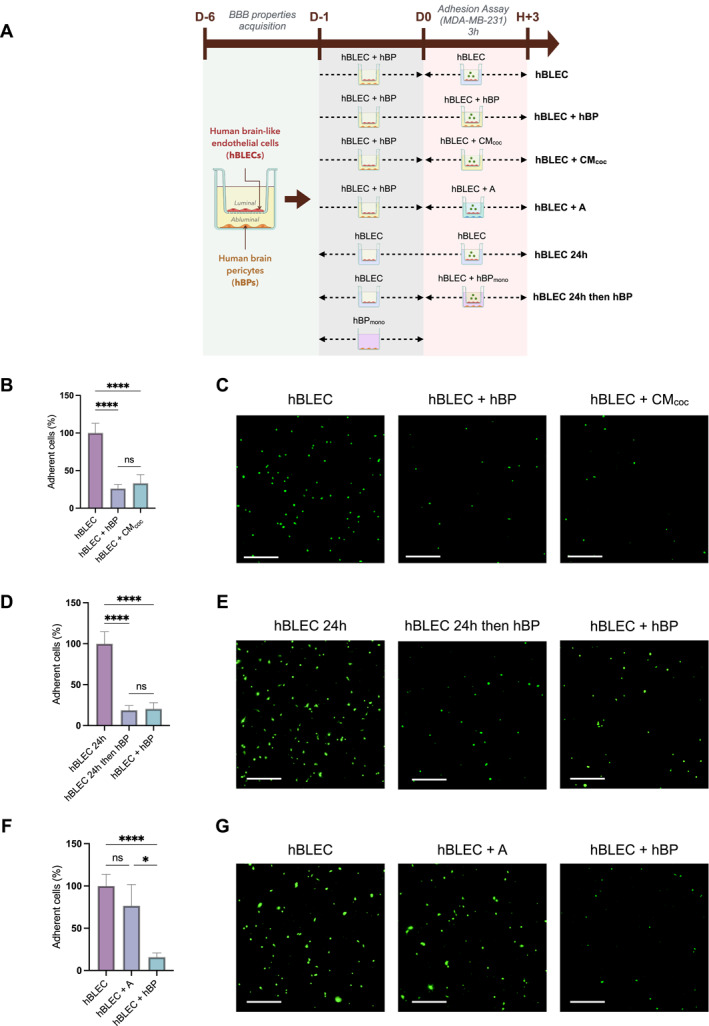
Brain pericyte secretions mediate the inhibition of TNBC cell adhesion to the BBB. (A) Experimental workflow for adhesion assay. All BBB experiments were performed using ECGMV2 medium with supplements for all conditions from day −6 to the end of the experiment. The astrocyte medium was renewed 24 h before the adhesion experiment with the same ECGMV2 medium. Endothelial cells (ECs) and brain pericytes (hBPs) were co‐cultured for 5 days, and inserts containing brain‐like endothelial cells (hBLECs) were then either maintained with hBPs or transferred to wells without hBPs. After 24 hours, and prior to MDA‐MB‐231 cells seeding, inserts were subjected to the following conditions: maintained without hBPs (hBLEC 24h); transferred to hBPs previously cultured alone for 24 hours (hBLEC 24h then hBP); transferred to wells without hBPs but containing either culture medium (hBLEC) or CM from co‐culture (hBLEC + CM_coc_); transferred to wells with astrocytes (hBLEC + A) or maintained in co‐culture with hBPs (hBLEC + hBP). (B–G), Quantification (B, D, F) of MDA‐MB‐231 cell adhesion to hBLECs after 3 h of incubation and representative images (C, E, G) of adhered MDA‐MB‐231 cells (green) under the different conditions. Data are obtained from three independent experiments, with three technical replicates per condition. Statistical analyses were performed using one‐way ANOVA followed by Tukey's test (B), Welch's ANOVA followed by Dunnett's T3 test (D) and Kruskal–Wallis test followed by Dunn's test (F). CM_coc_ = conditioned medium from 24 h hBLECs and hBPs coculture, A = astrocytes, hBP_mono_ = 24 h hBPs monoculture. Scale bar = 750 µm. BBB, blood–brain barrier; ECGMV2, EC Growth Medium MV 2; ECs, endothelial cells; hBLECs, human brain‐like endothelial cells; ns, non‐significant; hBPs, human brain pericytes; TNBC, triple‐negative breast cancer. ****p ≤ 0.0001; *p ≤ 0.05.

To investigate the role of hBPs in the development of TNBC brain metastases, adhesion of TNBC cells to hBLECs was analyzed. A human in vitro BBB model was used, in which hBLECs were co‐cultured with hBPs on insert filters, allowing hBLECs to be easily separated from hBPs for different incubation times (Figure 1A).

The presence of hBPs during adhesion assay significantly reduced MDA‐MB‐231 adhesion to the BBB by approximately 70% (Figure 1B,C) compared with hBLECs alone. A similar effect was observed for BT‐549 cells, another TNBC cell line (Figure S1A). Notably, the inhibitory effect on TNBC cell adhesion persisted when the adhesion assay was performed using CM collected from the co‐culture (Figure 1B,C) or hBPs monoculture (Figure S1B). These results suggest that soluble factors secreted by hBPs mediate this effect. Further, when adhesion assay was performed using basal medium (devoid of serum and supplements), TNBC cell adhesion to the BBB was reduced by approximately 50% in the presence of hBPs compared to the condition without hBPs (Figure S1C). This indicates that the inhibitory effect is not merely due to nutrient depletion but is instead attributable to the secretion of inhibitory factor(s) by hBPs. In this context, the content of CM from the abluminal compartment was analyzed using a cytokine array, and 24 soluble factors enriched in the presence of hBPs relative to hBLECs alone were identified (Figure S1D), and may contribute as signaling molecules to limit the adhesion of TNBC cells to the BBB. Importantly, when hBLECs were separated from hBPs for 24 h before the experiment and then transferred back to hBPs during the adhesion assay, TNBC cell adhesion to the BBB was reduced by 80% compared to the condition in which hBLECs were maintained without hBPs (Figure 1D,E). This decrease was comparable to the levels measured under continuous co‐culture between hBLECs and hBPs, highlighting the rapid and reversible nature of the hBP‐mediated inhibitory effect on TNBC cell adhesion. Interestingly, whereas hBPs markedly reduced TNBC cell adhesion to the BBB, astrocytes did not significantly reduce adhesion levels (Figure 1F,G).

### 3.2 Brain pericytes protect the endothelium from TNBC cells‐induced damage

**FIGURE 2:**
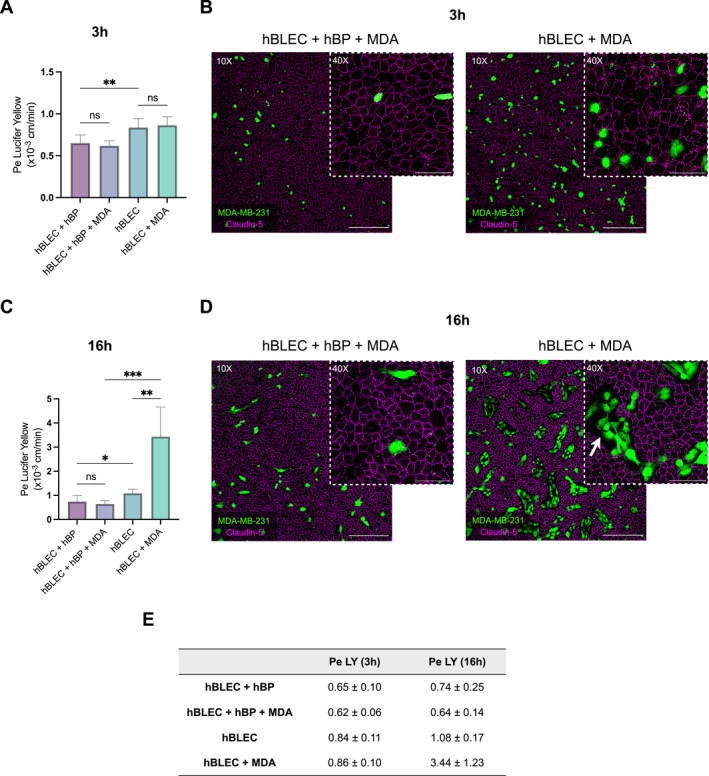
Brain pericytes maintain BBB integrity in the presence of TNBC cells. (A–E) Endothelial permeability (Pe) to Lucifer Yellow assessed after 3 h (A) or 16 h (C) of incubation with MDA‐MB‐231 cells, in the presence or absence of brain pericytes (hBPs). Permeability was also measured in the absence of cancer cells as a control. Permeability values (expressed in × 10^−3^ cm/min ± SD) are summarized in a table (E). Representative images of endothelial Claudin‐5 (magenta) immunostaining in the absence or presence of hBPs after 3 h (B) or 16 h (D) of incubation with MDA‐MB‐231 cells (green). Data are obtained from three independent experiments, with three technical replicates per condition. Statistical analyses were performed using one‐way ANOVA followed by Tukey's test (A) and Welch's ANOVA followed by Dunnett's T3 test (B). MDA = MDA‐MB‐231 cells. Scale bars = 300 μm (10X) and 100 μm (40X). BBB, blood–brain barrier; hBPs, human brain pericytes; ns, non‐significant; TNBC, triple‐negative breast cancer. ***p ≤ 0.001; **p ≤ 0.01; *p ≤ 0.05.

To ensure that the observed variation in TNBC cell adhesion previously observed was not due to disruption of the endothelial monolayer, the physical integrity of the BBB was assessed. First, endothelial permeability to Lucifer Yellow, a small hydrophilic molecule used as an integrity marker, was measured in the absence of cancer cells. We observed a 1.3‐fold increase in endothelial permeability when hBLECs were separated from hBPs for 3 h (Figure 2A,E).

Despite this slight increase in permeability, continuous Claudin‐5 staining, a key component of tight junctions, was maintained (Figure S2A). Interestingly, the addition of TNBC cells did not induce an increase in permeability, regardless of the presence or absence of hBPs for 3 h of incubation (Figure 2A,E). A continuous Claudin‐5 staining was also observed in these conditions (Figure 2B), indicating that the previous variations in adhesion were not due to the disruption of BBB tight junction staining caused by hBPs removal. However, when the incubation time was extended to 16 h, whereas permeability was preserved in the presence of hBPs, TNBC cells induced a 3.2‐fold increase in endothelial permeability only in the absence of hBPs (Figure 2C,E). Consistent with the permeability data, Claudin‐5 staining remained continuous in all conditions except when hBLECs were exposed to TNBC cells in the absence of hBPs (Figure 2D, Figure S2A), where areas lacking EC coverage were observed specifically at sites of cancer cell localization (Figure S2B). These findings suggest that hBPs exert a protective effect on the endothelial barrier against TNBC‐induced damage. This effect persists even after 48 h of co‐incubation with cancer cells (Figure S3).

### 3.3 Brain pericytes limit the hypoxia‐induced increase in TNBC cell adhesion to the endothelium and prevent endothelial integrity disruption by cancer cells under hypoxic conditions

**FIGURE 3:**
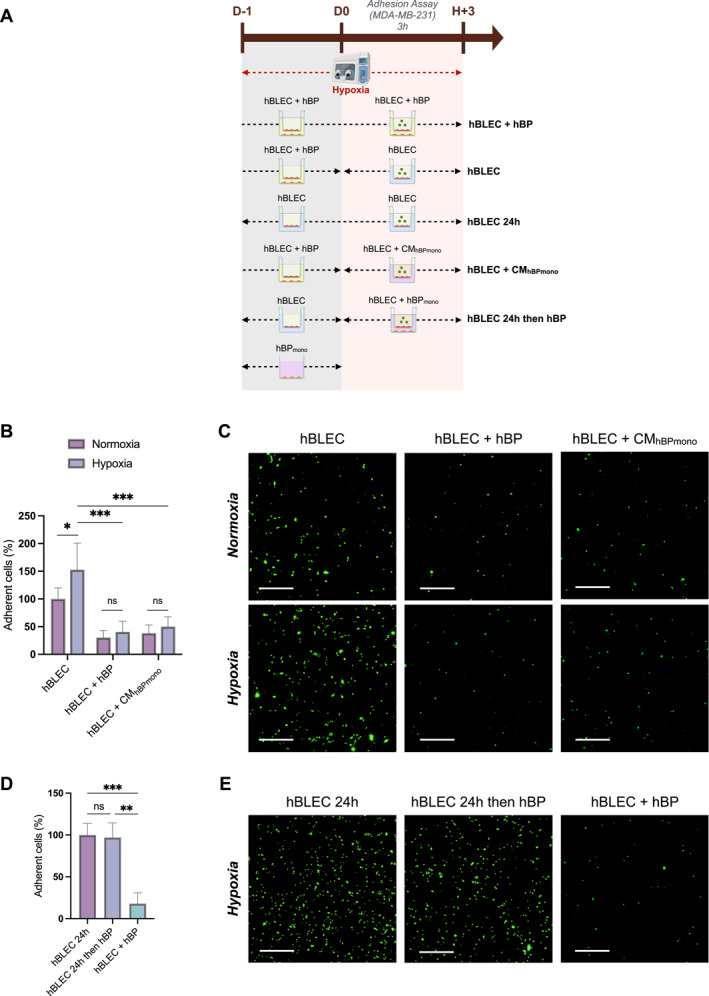
Brain pericytes limit TNBC cell adhesion to the BBB under hypoxic conditions. (A) Experimental workflow for adhesion assay in hypoxic conditions. All BBB experiments were performed using ECGMV2 medium with supplements for all conditions from day −6 to the end of the experiment. After 5 days of co‐culture with brain pericytes (hBPs), inserts containing brain‐like endothelial cells (hBLECs) were exposed to hypoxia for 24 h in the presence or absence of hBPs. Parallel conditions maintained in normoxia served as controls. (B–E) Quantification (B, D) of MDA‐MB‐231 cell adhesion to hBLECs after 3 h of incubation and representative images (C, E) of adhered MDA‐MB‐231 cells (green) under the different conditions. Data are obtained from three independent experiments, with three technical replicates per condition. Statistical analyses were performed using Welch's ANOVA followed by Dunnett's T3 test (B) and Kruskal‐Wallis test followed by Dunn's test (D). hBP‐CM_mono_ = conditioned medium from 24 h hBPs monoculture. Scale bar = 750 μm. BBB, blood–brain barrier; ECGMV2, EC Growth Medium MV 2; hBLECs, human brain‐like endothelial cells; hBPs, human brain pericytes; ns, non‐significant; TNBC, triple‐negative breast cancer. ***p ≤ 0.001; **p ≤ 0.01; *p ≤ 0.05.

To decipher if hypoxia influences TNBC cell interactions with hBLECs, BBB models were exposed to hypoxia for 24 h, followed by co‐incubation with TNBC cells under hypoxic conditions for 3 h (Figure 3A).

Under hypoxia and without hBPs, TNBC cell adhesion to the BBB increased by 50% compared to normoxic conditions (Figure 3B,C). However, no significant difference in adhesion was observed under hypoxia compared to normoxic conditions when hBPs were present, or when CM from hBPs was used. Notably, although a slight increase in endothelial permeability was observed under hypoxia, Claudin‐5 staining remained continuous in the presence and absence of hBPs for 3 h, similar to that observed under normoxia (Figure S4A–C). Nevertheless, in contrast to normoxia, under hypoxia, transient removal of hBPs for 24 h, followed by co‐culturing again with hBPs during adhesion assay, failed to inhibit TNBC cell adhesion (Figure 3D,E). Interestingly, in the absence of TNBC cells, a 4.2‐fold increase in endothelial permeability was observed under hypoxia without hBPs for 24 h, compared to normoxia, and was associated with tight junction destabilization and appearance of gaps in the endothelial monolayer (Figure S4A–C). This suggests that 24‐h exposure to hypoxia induces damage to the hBLECs in the absence of hBPs. Hence, hBPs maintain their capacity to inhibit TNBC cell adhesion to the BBB under hypoxia, with inhibition levels equivalent to normoxia. However, hBPs are unable to counteract the alterations induced by hypoxia on hBLECs that have been cultured without hBPs for 24 h.

**FIGURE 4:**
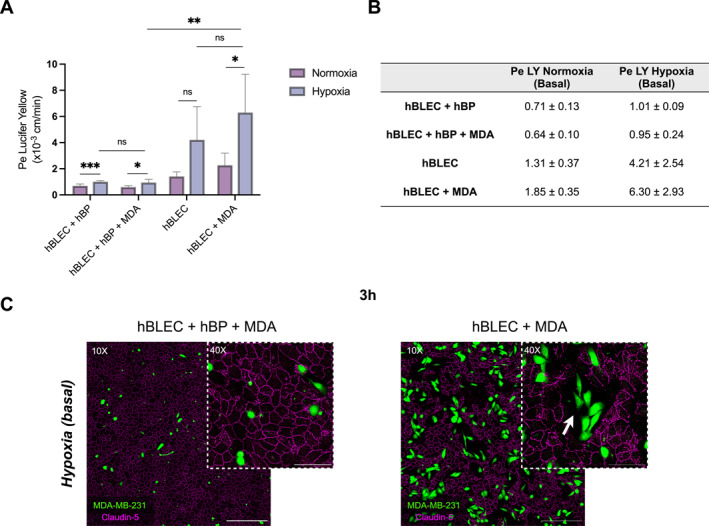
Brain pericytes maintain BBB integrity in the presence of TNBC cells under hypoxic conditions and supplement deprivation. (A) After 5 days of co‐culture with brain pericytes (hBPs) in ECGMV2 medium with supplements, inserts containing brain‐like endothelial cells (hBLECs) co‐cultured with hBPs were exposed to hypoxia for 16 h following medium replacement with ECGMV2 basal medium (without supplements). Then, endothelial permeability (Pe) to Lucifer Yellow was assessed after 3 h of incubation with MDA‐MB‐231 cells, in the presence or absence of hBPs, under normoxic and hypoxic conditions. (B) Permeability values (expressed in x10^−3^ cm/min ± SD) are summarized in a table. (C) Representative images of endothelial Claudin‐5 (magenta) immunostaining in the absence or presence of hBPs after 3 h of incubation with MDA‐MB‐231 cells (green) under hypoxic conditions in basal medium. Data are obtained from three independent experiments, with three technical replicates per condition. Statistical analyses were performed using Welch's ANOVA followed by Dunnett's T3 test. MDA = MDA‐MB‐231 cells. Scale bars = 300 μm (10X) and 100 μm (40X). BBB, blood–brain barrier; ECGMV2, EC Growth Medium MV 2; hBLECs, human brain‐like endothelial cells; hBPs, human brain pericytes; ns, non‐significant; TNBC, triple‐negative breast cancer. ***p ≤ 0.001; **p ≤ 0.01; *p ≤ 0.05.

We next evaluated the impact of TNBC cells on endothelial integrity under hypoxic and nutrient‐deprived conditions, in the presence or absence of hBPs during the 3‐h adhesion assay. In the presence of hBPs, TNBC cells do not induce a significant change in endothelial permeability under hypoxia (Figure 4A,B).

In contrast, in the absence of hBPs, TNBC cells induce a 6.6‐fold increase in permeability compared to the condition with hBPs. Furthermore, endothelial permeability was 3.4‐fold higher in the presence of TNBC cells under hypoxia in the absence of hBPs than under normoxia. Consistently, although tight junctions staining remained continuous under hypoxia and nutrient deprivation in the presence of hBPs or when HBLECs were incubated with co‐culture CM, the absence of hBPs led to tight junction destabilization and the formation of gaps in the endothelium monolayer (Figure S4D). Notably, the disruption of Claudin‐5 staining under hypoxia and nutrient deprivation in the absence of hBPs appeared more pronounced following TNBC cells incubation, with larger areas lacking ECs, while continuous tight junction staining was observed in the presence of hBPs (Figure 4C). Together, these findings indicated that hypoxia combined with nutrient deprivation compromises endothelial barrier function and sensitizes the BBB to TNBC cell‐induced damage, an effect that is prevented by hBPs.

### 3.4 Brain pericytes enhance aggressiveness of TNBC cells

Following transendothelial migration, TNBC cells are likely to interact directly with pericytes in the brain parenchyma. After demonstrating the protective role of hBPs on hBLECs from TNBC cells, we next investigated whether hBPs also influence properties of TNBC cells themselves.

**FIGURE 5:**
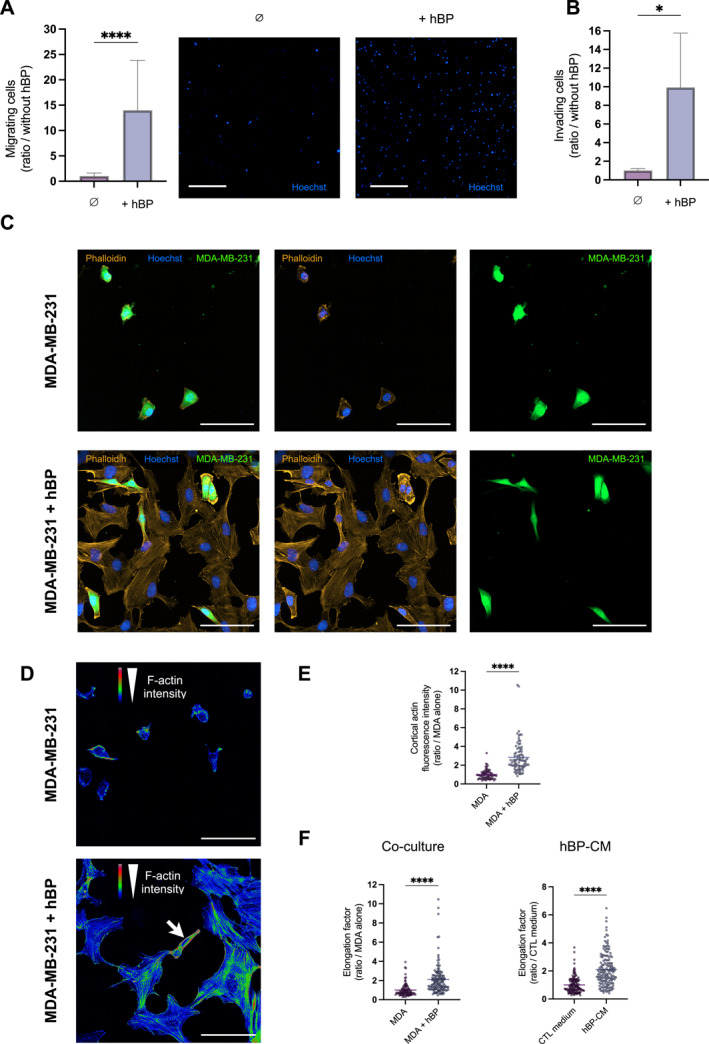
Brain pericytes enhance migratory and invasion properties of TNBC cells. (A) Quantification of MDA‐MB‐231 cells that migrated to the lower side of insert filters in the absence (∅) or presence of brain pericytes (+hBPs), and representative images of migrated cells (nuclei stained in blue) associated. (B) Quantification of MDA‐MB‐231 cells that invaded the lower side of insert filters pre‐coated with Matrigel^®^ in the presence (+hBP) or absence (∅) of hBPs. (C) Representative images of MDA‐MB‐231 cells (green) after 48 h of monoculture or co‐culture with hBPs; cell morphology is visualized by F‐actin staining (phalloidin, orange). (D) Heatmap visualization of F‐actin intensity. (E) Quantification of cortical F‐actin fluorescence in MDA‐MB‐231 cells. (F) MDA‐MB‐231 cell elongation factor, defined as the ratio of cell length to width, following 48 h of monoculture (MDA) and co‐culture with hBPs (MDA + hBP), or following 1‐hour treatment with DMEM 0.1% (CTL medium) and hBP‐CM. Data are obtained from three independent experiments, with three technical replicates (A, B) or at least 30 cells analyzed (E, F) per condition. Statistical analyses were performed using a Mann–Whitney test (A, E, F) and an unpaired *t*‐test with Welch's correction (B). MDA = MDA‐MB‐231 cells. Scale bars = 750 μm (A) and 100 μm (C, D). DMEM, Dulbecco'’s Modified Eagle Medium; hBP‐CM, hBP‐conditioned medium; hBPs, human brain pericytes; TNBC, triple‐negative breast cancer. ****p ≤ 0.0001; *p ≤ 0.05.

The impact of hBPs on TNBC cell migration and invasion was first assessed. A 14‐fold increase in migratory capacity (Figure 5A) and a 10‐fold increase in invasion were observed (Figure 5B) when TNBC cells were co‐cultured with hBPs.

Beyond paracrine signaling, TNBC cells were also found to establish direct contacts with hBPs (Figure 5C). Interestingly, after 48 hours of co‐culture with hBPs, cortical F‐actin fluorescence intensity was increased in TNBC cells compared to monoculture, indicating cytoskeletal remodeling driven by hBPs (Figure 5C–E). In parallel, both direct co‐culture with hBPs and exposure to hBP‐CM led to a more elongated TNBC cell morphology compared to the monoculture condition (Figure 5C,F), further supporting a role for hBPs in promoting a migratory morphology for TNBC cells.

**FIGURE 6:**
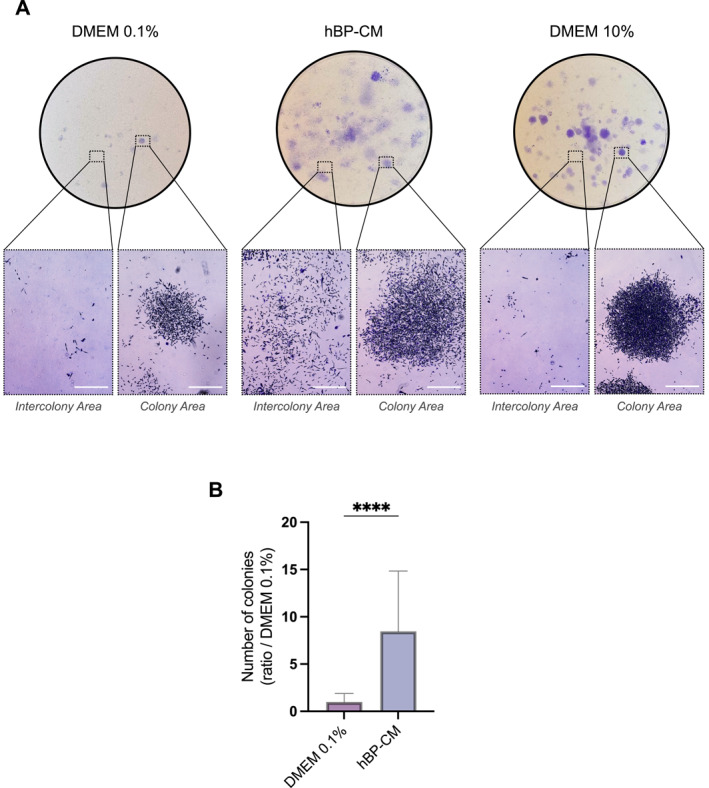
Brain pericyte secretions enhance TNBC clonogenic capacity. (A) Representative images of MDA‐MB‐231 colony formation following exposure to conditioned medium from brain pericytes (hBP‐CM), control medium (DMEM 0.1%, medium used for CM generation), or DMEM 10%. (B) Quantification of colony numbers formed by MDA‐MB‐231 under previously indicated conditions. Data are obtained from three independent experiments, with three technical replicates per condition. Statistical analyses were performed using a Mann–Whitney test. MDA = MDA‐MB‐231 cells. Scale bar = 600 μm. DMEM, Dulbecco'’s Modified Eagle Medium; hBP‐CM, hBP‐conditioned medium; TNBC, triple‐negative breast cancer.

We then examined whether hBPs also influence the capacity of TNBC to form colonies from single cells using clonogenic assays. TNBC cells exposed to hBP‐CM formed an 8‐fold higher number of colonies than those cultured with the control medium (Figure 6A,B).

Notably, colonies in the hBP‐CM condition appeared more diffused, with increased dispersion of individual TNBC cells between colonies, in contrast to the more delineated colonies observed in the DMEM 10% condition (Figure 6A).

Taken together, these findings demonstrate that hBPs enhance TNBC cell migration, invasion, and clonogenic capacity, suggesting that hBPs may promote a more aggressive phenotype for TNBC cells and may facilitate metastatic outgrowth within the brain microenvironment.

## 4 DISCUSSION

**FIGURE 7:**
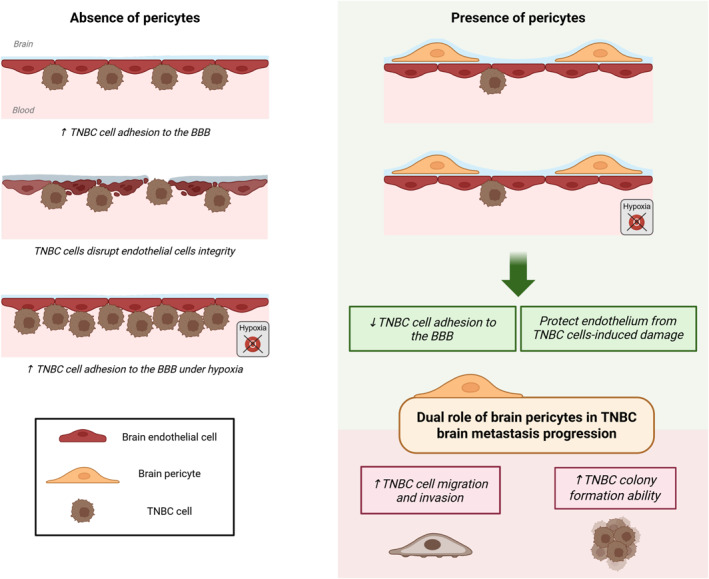
Schematic overview of the dual role of brain pericytes in TNBC brain metastasis development. The figure was created with *Biorender.com*. TNBC, triple‐negative breast cancer.

Pericytes are mural cells predominantly distributed around microvessels and capillaries. In the CNS, pericyte coverage is particularly high, with an EC‐to‐pericyte ratio varying from 1:1 to 3:1.^22^ Brain pericytes play a pivotal role in establishing and preserving BBB properties, thereby contributing to the maintenance of brain homeostasis. In the context of breast cancer brain metastasis, alterations in the pericytes population have been observed, characterized by a reduction in CD31+ pericytes and an increase in Desmin + pericytes, correlating with enhanced BBB permeability.^23^ In this study, we investigated the role of hBPs in TNBC brain metastasis homing and development (Figure 7).

We first demonstrated that hBPs, but not astrocytes, limit TNBC cell adhesion to the BBB, without altering its physical integrity. These findings are consistent with previous work demonstrating that co‐culturing ECs with pericytes, but not with astrocytes, reduced lung cancer cell colony formation on the endothelium compared with endothelium alone.^24^ The increased adhesion of cancer cells observed in the absence of hBPs may result from a direct effect of pericyte secretions on TNBC cells and/or from effects on ECs, such as the upregulation of endothelial adhesion molecules. Prior studies have shown that pericytes modulate endothelial ICAM‐1 and VCAM‐1 expression, key proteins implicated in cell‐cell adhesion, with the absence of pericytes leading to increased levels of these proteins.^11^, ^25^, ^26^, ^27^ One proposed mechanism involves pericytes‐secreted Angiopoietin‐1 binding to endothelial Tie2, activating ABIN (A20‐binding inhibitor of NF‐κB), which inhibits NF‐κB signaling and thereby reduces ICAM‐1 and VCAM‐1 expression.^28^, ^29^ In this study, hBPs exerted a rapid inhibitory effect on adhesion through the secretion of soluble factors, potentially by regulating the membrane exposure of specific adhesion molecules. We found a higher abundance of soluble VCAM‐1 in the CM of the abluminal compartment in the presence of hBPs compared to hBLECs alone. VCAM‐1, usually a membrane protein, can be released as soluble fragments through proteolytic cleavage^30^ that have been reported to inhibit leukocyte adhesion to the endothelium.^31^ Hence, hBPs may modulate the proteolytic shedding of VCAM‐1, which could explain the decrease in TNBC cell adhesion observed in the presence of hBPs by potentially reducing VCAM‐1 interactions with integrins on cancer cells. Moreover, we also identified by cytokine array increased levels of Pentraxin‐3, GDF‐15, and IGFBP3, which have been reported to reduce the adhesion of immune cells to ECs.^32^, ^33^, ^34^ Further analyses will be required to precisely identify the secreted factors involved. Pericytes are also implicated in maintaining the endothelial glycocalyx, a surface layer of glycoproteins, proteoglycans, and glycosaminoglycans that limits exposure of membrane proteins, including adhesion molecules such as ICAM‐1 and VCAM‐1, thereby reducing cell adhesion. In pericytes‐deficient mice, genes essential for glycocalyx maintenance are downregulated, resulting in reduced brain endothelial glycocalyx coverage.^26^

Although separating hBLECs from hBPs only slightly increased BBB permeability, tight junction staining remained continuous in the absence of TNBC cells. Pericytes are known to regulate not only endothelial junctions but also vesicular trafficking; their absence increases endothelial transcytosis,^11^ which may explain the elevated permeability observed without visible Claudin‐5 staining disruption in our study. Several pericytes‐secreted factors, such as Angiopoietin‐1 and GDNF, enhance expression of tight junction proteins, including Claudin‐5, ZO‐1, and Occludin.^35^, ^36^, ^37^ In the presence of TNBC cells, endothelial barrier integrity is preserved during short‐term conditions (3 h) but is disrupted after 16 h, specifically in the absence of hBPs. A previous in vitro study using hBMECs (human brain microvascular ECs) without pericytes similarly reported that MDA‐MB‐231 cells increased endothelial permeability.^38^ These findings suggest that omission of pericytes in BBB models may introduce experimental bias, and could explain the rapid and severe alteration of EC integrity by TNBC cancer cells observed in a previous study conducted in the absence of brain pericytes.^39^

In the present study, hypoxia enhances TNBC cell adhesion to the BBB and damage to the endothelium in the absence of hBPs, effects that were prevented in their presence. This is consistent with a study using HUVECs (human umbilical vein endothelial cells), where hypoxia increased ICAM‐1 expression and enhanced their interactions with monocytes.^40^ By inducing cellular stress, hypoxia increases adhesion to the endothelium in the absence of hBPs, suggesting that hBPs may secrete factors that protect ECs from this stress. However, a direct effect of hBPs on TNBC cells to reduce BBB adhesion cannot be excluded. Hypoxic regions have been observed in mouse models of breast cancer brain metastasis, associated with vascular occlusion by cancer cells and a consequent increase in brain colonization mediated by Angiopoietin‐2 and VEGF.^10^ Hypoxia can also regulate BBB properties. Studies conducted in the absence of pericytes have reported that hypoxia disrupts endothelial integrity,^41^, ^42^, ^43^ which is in agreement with our results. Our findings are also consistent with previous studies that indicate that hBPs protect against hypoxia‐induced BBB disruption.^44^, ^45^ In contrast, HIF‐1 stabilization in pericytes has also been reported to disrupt endothelial integrity.^46^ Other studies report that hypoxia could enhance BBB properties.^47^, ^48^ This difference may be due to the use of bovine ECs^48^ instead of human ones, or from hypoxia exposure occurring during the differentiation process^47^ rather than after, as in our study. Moreover, in vivo data have demonstrated that pericyte loss itself can induce hypoxia,^26^ highlighting the need to investigate in the future whether alterations in pericyte subpopulations during breast cancer brain metastasis^23^ contribute to the development of a hypoxic microenvironment.

Beyond their BBB‐protective roles against cancer cell adhesion during the early stages of brain metastasis development, our work demonstrates that hBPs also promote pro‐metastatic behaviors in TNBC cells, which may facilitate their progression into the brain parenchyma at later stages. It was previously reported that breast cancer cells preferentially migrate toward pericytes rather than ECs.^49^ Our data support these findings, where TNBC cell migration was enhanced in the presence of hBPs compared to a control medium. Pericytes can also promote breast cancer cell proliferation through IGF‐2 signaling and enhance cancer cell adhesion to the culture dish surface, likely through the increased secretion of some extracellular matrix components, fibronectin and collagen IV, which provide a supportive adhesive substrate.^49^ The combination of improved adhesion to the culture dish, increased proliferation, and elevated migratory capacity induced by hBP‐CM likely explains the more diffuse colonies and the increased number of individual cells dispersed between colonies. Further investigations are necessary to elucidate the mechanisms underlying TNBC cell migration and invasion induced by hBPs, particularly the potential roles of increased cancer cell elongation and cortical actin remodeling. The regulation of F‐actin polymerization at membrane protrusions is a critical step in cell migration,^50^ and enhanced cell elongation has been correlated with increased migratory capacity.^51^ Additionally, fibronectin and collagen IV, both abundantly secreted by pericytes,^49^ have been shown to modulate the elongation of MDA‐MB‐231 cells.^51^

## 5 CONCLUSION

Collectively, this study reveals a dual role for hBPs in TNBC brain metastasis. They limit initial adhesion of TNBC cells to the BBB and protect the endothelium from cancer cell‐induced damage, while also enhancing the TNBC cell migration, invasion, and colony formation ability when the BBB is crossed. Pericytes coverage emerges as a critical determinant of vascular integrity in the context of brain metastasis and under hypoxic stress. Age,^52^ certain therapies,^53^ and pathological conditions^54^, ^55^, ^56^ can reduce pericyte coverage, potentially increasing susceptibility to brain metastasis. Emerging evidences highlight brain pericytes as a promising therapeutic target in TNBC brain metastasis.^57^ A deeper mechanistic understanding of both the BBB‐protection against TNBC cells as well as the pro‐metastatic role of brain pericytes will be essential for developing targeted therapeutic strategies. Strategies that enhance BBB protection against TNBC cells or limit brain pericytes‐mediated tumor progression within the brain parenchyma may help improve the poor prognosis of patients with TNBC brain metastases.

## AUTHOR CONTRIBUTIONS

E.H. performed the experiments; M.Ca. and J.C. provided technical support in cell culture and data analysis, L.D. provided training in cell culture protocols, E.H., R.‐A.T. and C.M. wrote the manuscript; R.‐A.T and C.M. conceived, designed the experiments; F.S., T.K., C.L. and X.L.B. provided resources; C.M. and R.‐A.T. provided resources and acquired fundings; C.M., R.‐A.T., F.G., M.Cu. managed the administrative part of the project; All authors provided critical review of the manuscript and have given approval to the final version of the manuscript.

## CONFLICT OF INTEREST STATEMENT

The authors declare no conflicts of interest.

## ETHICS STATEMENT

Human pericytes are from the cell line named hBPs and has been provided by Professor Takashi Kanda's group from Yamagushi university (Japan). The study protocol was approved by local ethic committee and conducted in accordance with the declaration of Helsinki, as amended in Somerset West in 1996 (23). The collection and use of CD‐34^+^ stem cells derived from cord blood has been approved since 2011 by the French ministry (CODECOH 2011‐1321). These cells were harvested thanks to an agreement established between the hospital of Béthune and university of Artois. Informed consents were obtained from the families before starting the study.

## Supporting information

Supporting Information S1

Figure S1

Figure S2

Figure S3

Figure S4

## Data Availability

All data generated or analyzed during this study are included in this article and/or its supplementary material files.
