# Supplementary material for: Human brain pericytes protect the blood–brain barrier from triple‐negative breast cancer cells while promoting tumor aggressiveness
Source: J Cell Commun Signal. 2026 May 3;20(2):e70070. doi: 10.1002/ccs3.70070 (PMC13135669; doi:10.1002/ccs3.70070)
Supplement: Supplementary file 1 — Supporting Information S1 [file CCS3-20-e70070-s003.docx]

**Cell culture**

The TNBC cell line BT-549 was obtained from the ATCC. TNBC cells were cultured in MEM (Gibco) supplemented with 10% (v/v) heat-inactivated FBS, 1% (v/v) non-essential amino acids (Gibco), 1% (v/v) penicillin-streptomycin (Gibco).

**Cytokine array:**

The relative expression of multiple cytokines in our BBB model was assessed using the Proteome Profiler Human XL Cytokine Array Kit (#ARY022B, R&D Systems), according to the manufacturer’s instructions. Briefly, cell culture supernatants were collected from the abluminal compartment of co-cultures containing hBLECs with hBPs or hBLECs alone, following a 3-hour incubation with MDA-MB-231 cells. The cellular supernatants from the three technical replicates of each condition were pooled and then centrifuged (1000 RPM, 10 min), and the resulting supernatants were stored at -80°C. Array membranes pre-spotted with capture antibodies were first blocked for 1 hour at room temperature on a rocking platform using the array buffer provided, then cell culture supernatants were incubated with the membranes (16h, 4°C). The next day, membranes were washed and incubated with Detection Antibody Cocktail for 1 hour at room temperature on a rocking platform. After washing, membranes were incubated with Streptavidin–Horseradish Peroxidase for 30 minutes at room temperature on a rocking platform. After final washes, signal detection was performed using chemiluminescent substrate and captured using a chemiluminescence imaging system (Fusion FX, Vilber). Densitometric analysis of individual cytokine spots from three independent experiments was performed using ImageJ software. Background signals were subtracted, and the intensity of each spot was normalized to the signal obtained from hBLECs cultured without hBPs.
