# Supplementary material for: Human brain pericytes protect the blood–brain barrier from triple‐negative breast cancer cells while promoting tumor aggressiveness
Source: J Cell Commun Signal. 2026 May 3;20(2):e70070. doi: 10.1002/ccs3.70070 (PMC13135669; doi:10.1002/ccs3.70070)
Supplement: Supplementary file 2 — Figure S1 [file CCS3-20-e70070-s002.pdf]

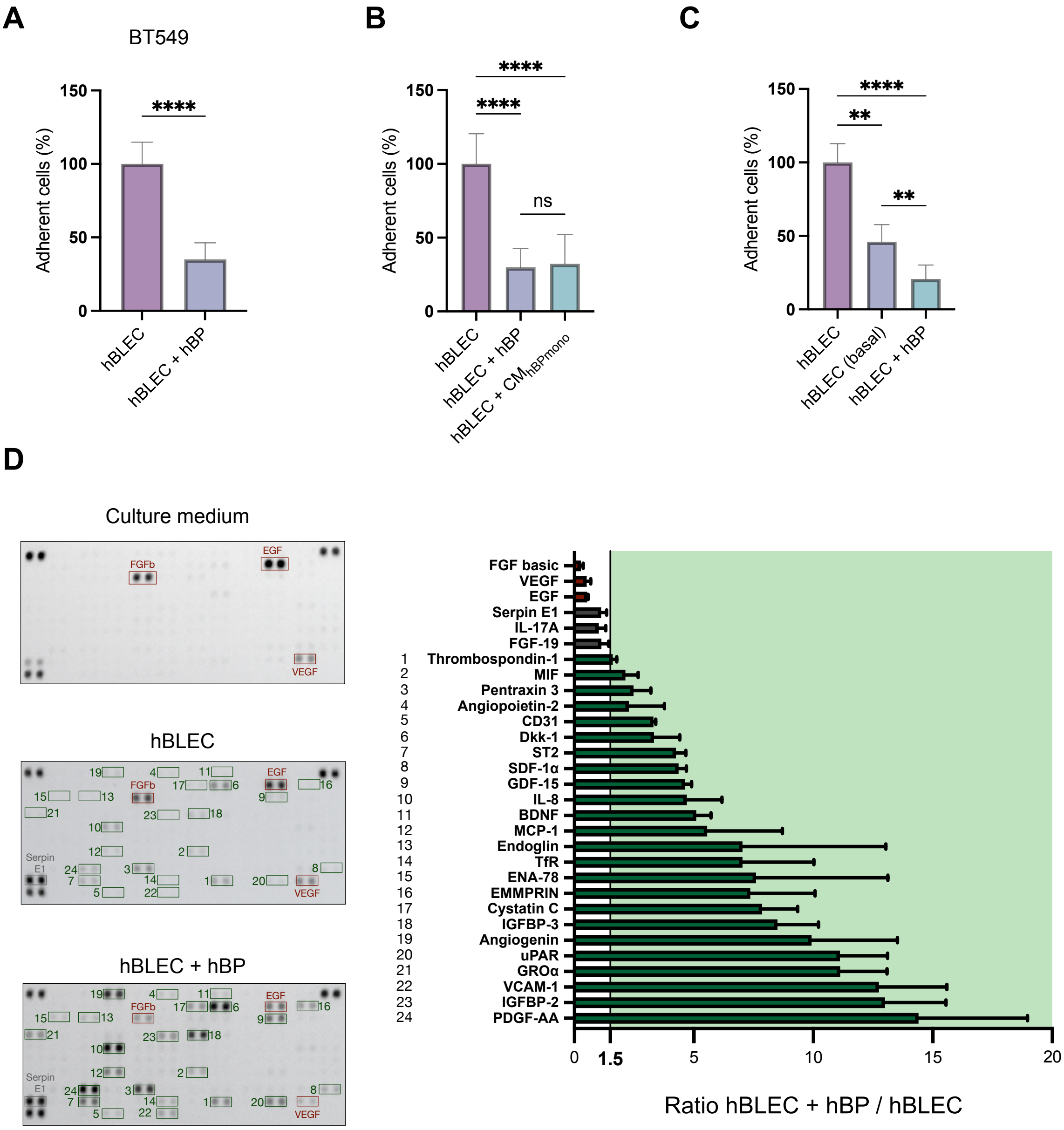

Figure S1: **Communication between endothelium and brain pericytes modulates TNBC cell adhesion through diffusible factors**. All BBB experiments were performed using ECGMV2 medium with supplements for all conditions, from day -6 to the end of the experiment. **A** Quantification of BT-549 cell adhesion to brain-like endothelial cells (hBLECs) after 3 hours of incubation in the absence or presence of brain pericytes (hBPs). **B** Quantification of MDA-MB-231 cell adhesion to hBLECs after 3 hours of incubation in the absence of hBPs with culture medium or with hBPs monoculture CM (hBLEC + $CM_{hBPmono}$). **C** Quantification of MDA-MB-231 cell adhesion to hBLECs after 3 hours of incubation in the absence of hBPs with

complete culture medium or basal culture medium (without supplements, EC basal). **D** Cytokine array analysis of CM collected from the abluminal compartment in the presence or absence of hBPs. The quantification associated is expressed as the ratio of signal intensity in the condition with hBPs to that in the condition without hBPs. Data are obtained from three independent experiments, with three technical replicates per condition. Statistical analyses were performed using unpaired t-test (**A**), one-way ANOVA followed by Tukey's test (**B**) and Kruskal-Wallis test followed by Dunn's test (**C**). _$CM_{hBPmono}$ = conditioned medium from 24h hBPs monoculture. ****$P \leq 0.0001$; **$P \leq 0.01$; ns, non-significant._
