# Supplementary material for: Human brain pericytes protect the blood–brain barrier from triple‐negative breast cancer cells while promoting tumor aggressiveness
Source: J Cell Commun Signal. 2026 May 3;20(2):e70070. doi: 10.1002/ccs3.70070 (PMC13135669; doi:10.1002/ccs3.70070)
Supplement: Supplementary file 3 — Figure S2 [file CCS3-20-e70070-s001.pdf]

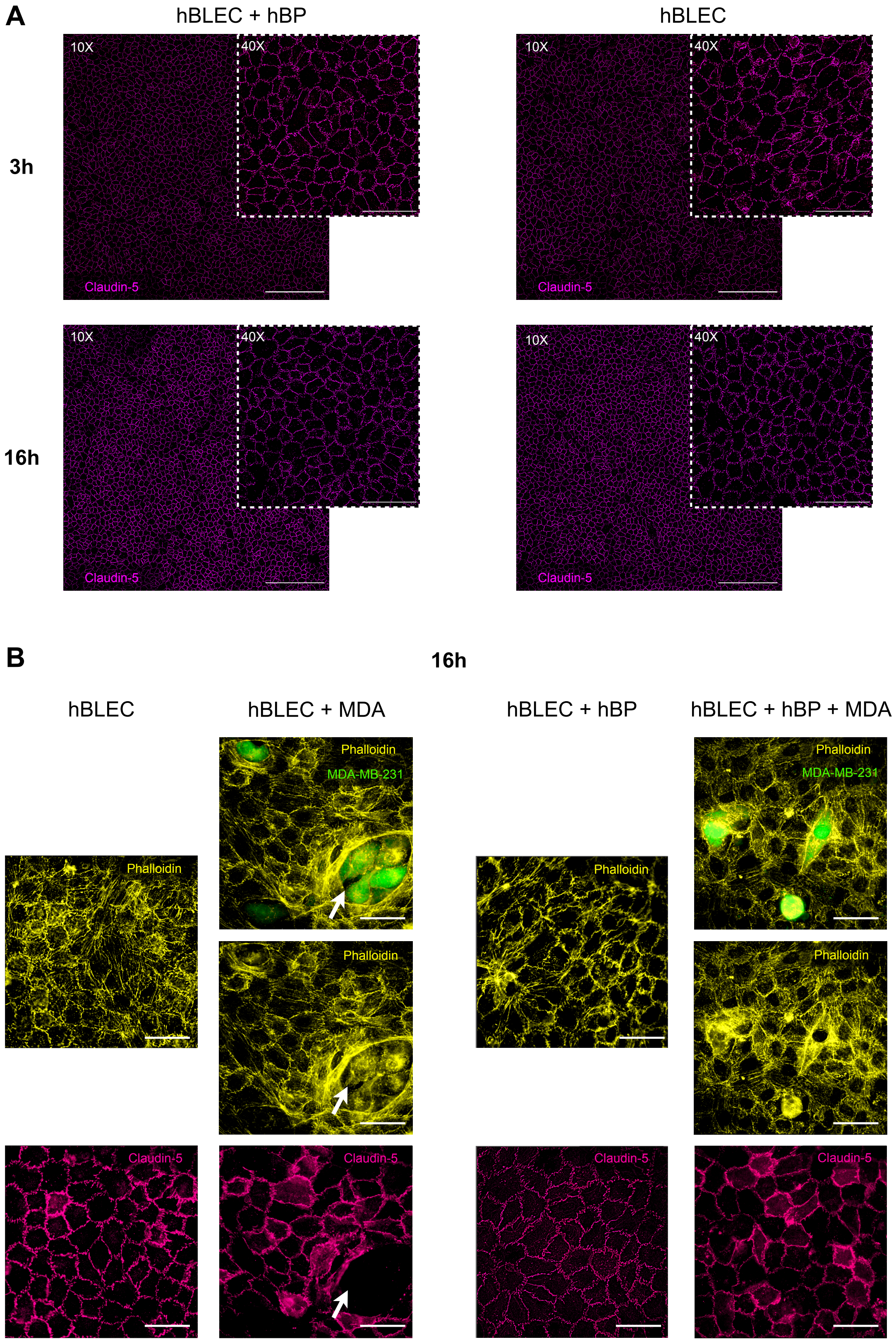

Figure S2: **Endothelial integrity is preserved without brain pericytes in the absence of TNBC cells**. **A** Immunostaining of endothelial Claudin-5 (magenta) in the absence or presence

of brain pericytes (hBPs) for 3 or 16 hours. **B** Analysis of hBLECs morphology by F-actin staining (phalloidin, yellow), in the absence or presence of MDA-MB-231 cells (green) for 16 hours. Images are representative of three independent experiments. *MDA = MDA-MB-231 cells. Scale bars = 300 μm (10X), 100 μm (40X) and 50 μm (**B**).*
