# Supplementary material for: Human brain pericytes protect the blood–brain barrier from triple‐negative breast cancer cells while promoting tumor aggressiveness
Source: J Cell Commun Signal. 2026 May 3;20(2):e70070. doi: 10.1002/ccs3.70070 (PMC13135669; doi:10.1002/ccs3.70070)
Supplement: Supplementary file 4 — Figure S3 [file CCS3-20-e70070-s005.pdf]

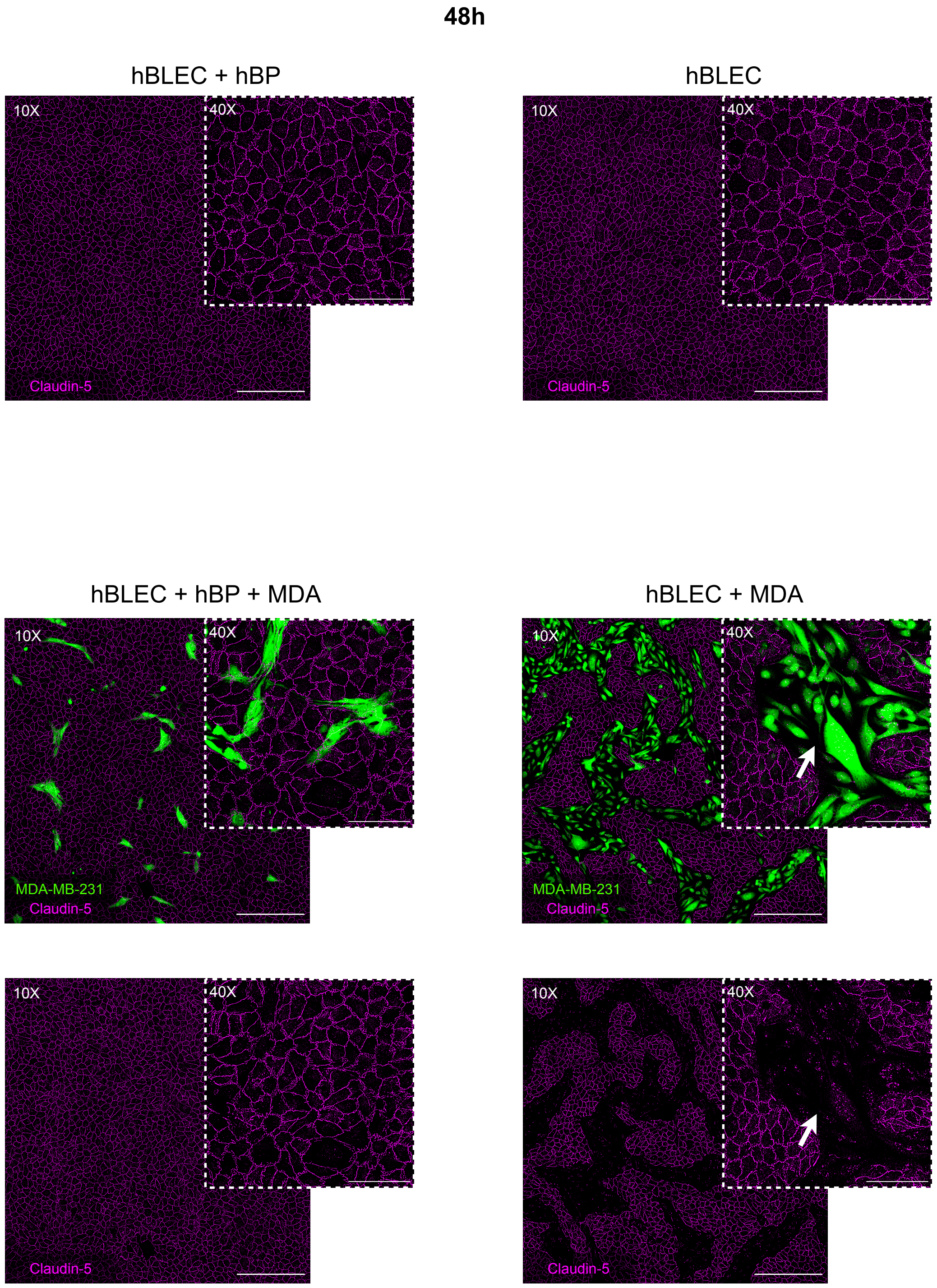

Figure S3: **Prolonged incubation with TNBC cells does not compromise endothelial integrity in the presence of brain pericytes.** Immunostaining of endothelial Claudin-5 (magenta) in the absence or presence of brain pericytes (hBPs) after 48 hours of incubation with MDA-MB-231 cells (green). Images are representative of three independent experiments. *MDA = MDA-MB-231 cells. Scale bars = 300 µm (10X), 100 µm (40X).*
