# Supplementary material for: Human brain pericytes protect the blood–brain barrier from triple‐negative breast cancer cells while promoting tumor aggressiveness
Source: J Cell Commun Signal. 2026 May 3;20(2):e70070. doi: 10.1002/ccs3.70070 (PMC13135669; doi:10.1002/ccs3.70070)
Supplement: Supplementary file 5 — Figure S4 [file CCS3-20-e70070-s004.pdf]

A

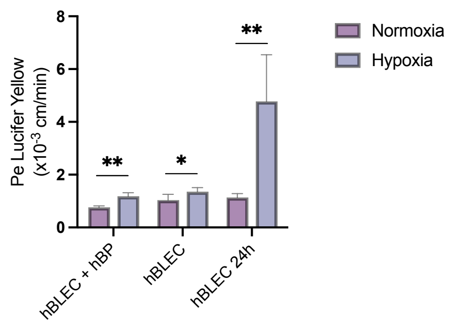

B

	Pe LY (Normoxia)	Pe LY (Hypoxia)
**hBLEC + hBP**	0.76 ± 0.06	1.18 ± 0.13
**hBLEC**	1.04 ± 0.22	1.35 ± 0.15
**hBLEC 24h**	1.14 ± 0.14	4.77 ± 1.77

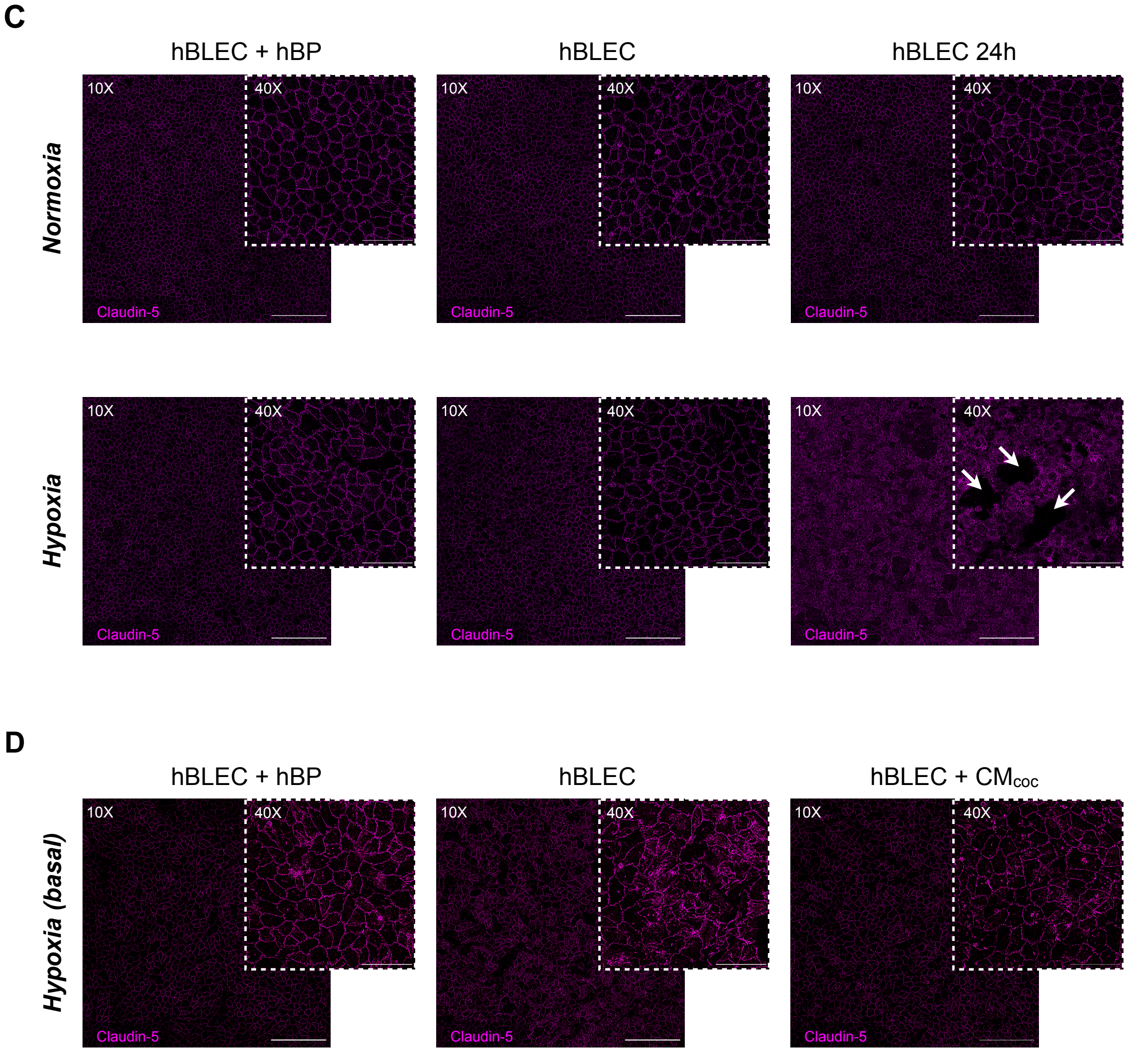

Figure S4: **Brain pericytes protect endothelium against hypoxia-induced damage. A** After 5 days of co-culture with brain pericytes (hBPs), inserts containing brain-like endothelial cells (hBLECs) were exposed to hypoxia for 24 hours in the presence or absence of hBPs. After this time, inserts containing hBLECs were transferred into wells without hBPs for 3 hours or maintained in the presence or absence of hBPs. Parallel conditions maintained in normoxia

served as controls. Then, endothelial permeability (Pe) to Lucifer Yellow was assessed. **B** Permeability values (expressed in x10^{-3} cm/min ± SD) are summarized in a table. **C** Immunostaining of endothelial Claudin-5 (magenta) in the presence or absence of hBPs for 3 or 24 hours under normoxic and hypoxic conditions in complete culture medium. **D** Representative images of endothelial Claudin-5 (magenta) immunostaining in the presence or absence of hBPs for 3 hours under hypoxic conditions in basal culture medium (without supplements). Data are obtained from three independent experiments, with three technical replicates per condition. Statistical analyses were performed using Kruskal-Wallis test followed by Dunn's test. *CM_{coc} = conditioned medium from 24 hours of hBLECs and hBPs coculture. **P ≤ 0.01; *P≤ 0.05. Scale bars = 300 μm (10X), 100 μm (40X).*
